# Eleven-Year Trends in Stillbirth, Neonatal and Infant Mortality and Birthweight-Specific Risk: A BABIES Matrix Analysis from a Tertiary Maternity Center in Kazakhstan, 2015–2025

**DOI:** 10.3390/children13101284

**Published:** 2026-09-22

**Authors:** Ardak Ayazbekov, Saken Khaidarov, Mukhabat Nartayeva, Venera Israilova, Assel Arginbekova, Aliya Aubakirova, Zhulduz Galiyeva, Aliya Meirmanova, Gulmira Rapilbekova, Dair Seisembekov

**Affiliations:** 1 Scientific Centre of Obstetrics, Gynaecology and Perinatology, Almaty 0500010, Kazakhstan; ardakayazbekov966@gmail.com (A.A.); muminovna@mail.ru (M.N.); aliya.aubakirova@mail.ru (A.A.); galieva140276@mail.ru (Z.G.); aliya.med@mail.ru (A.M.); 2 Department of Molecular Biology and Medical Genetics, Asfendiyarov Kazakh National Medical University, Almaty 050004, Kazakhstan; 3 Department of Cardiology, City Cardiology Centre, Faculty of Medicine, University of International Business, Abay Ave. 8A, Almaty 050010, Kazakhstan; israilova.venera@mail.ru; 4 Corporate Fund “University Medical Center”, Astana 010000, Kazakhstan; gulmirarapilbek@gmail.com; 5 Center of Obstetrics, Gynecology and Neonatology, National Coordination Center for Emergency Medicine, Astana 010000, Kazakhstan

**Keywords:** stillbirth, neonatal mortality, infant mortality, perinatal mortality, birthweight, BABIES matrix, perinatal periods of risk, Kazakhstan, low birthweight, quality of care

## Abstract

**Highlights:**

**What are the main findings?**

- Over eleven years, infant mortality halved, and the stillbirth rate fell by almost half, but the intrapartum component did not follow: no clear change was detected between 2015 and 2025 (rate ratio 0.94, 95% CI 0.38–2.32).
- Births below 2500 g were 5.9% of all births yet accounted for 71.1% of composite feto-infant deaths, and decomposition placed the decline within birthweight bands rather than in a shift of the birthweight distribution.

**What are the implication of the main findings?**

- The BABIES matrix turns routine facility statistics into an explicit allocation of attention and can be run where patient-level linkage is unavailable, which makes it usable for perinatal surveillance across much of Central Asia.
- Intrapartum stillbirth and the group below 2500 g are where any further reduction would have to be found; at this volume, both are small enough for case-by-case audit, which an ecological series cannot replace.

**Abstract:**

**Background/Objectives:** Long-run facility-level perinatal data from Central Asia are scarce. We described eleven years of stillbirth, neonatal, infant and perinatal mortality at one tertiary maternity center in Kazakhstan, and used the Birthweight and Age-at-death Boxes for Intervention and Evaluation System (BABIES) matrix to locate where feto-infant deaths concentrated. **Methods:** Retrospective ecological analysis of aggregated annual institutional records, 2015–2025: 102,481 total births, 101,544 live births, 937 stillbirths and 489 infant deaths. Rates were modeled by Poisson regression with the log birth denominator as offset, refitted as quasi-Poisson and negative binomial. Composite feto-infant mortality (antenatal and intrapartum stillbirth plus death at 0–27 days, per 1000 total births in each birthweight band) was examined by direct standardization and Kitagawa decomposition, using the nine years whose matrix cells reconciled with the registry. The study was not preregistered. **Results:** Infant mortality fell from 7.07 to 3.57 per 1000 live births (rate ratio 0.50, 95% CI 0.33–0.77) and stillbirth from 10.07 to 5.42 per 1000 total births (0.54, 0.38–0.76), an average annual decline near 8% for neonatal and infant mortality. Antenatal stillbirth declined; no clear change was detected for intrapartum stillbirth (1.11 to 1.04 per 1000; 0.94, 0.38–2.32). Births below 2500 g were 5.9% of all births but 71.1% of composite feto-infant deaths (rate ratio 39). Decomposition placed the reduction within birthweight bands rather than in the birthweight distribution, in single-year and pooled-period versions. **Conclusions:** The design is ecological and descriptive: it shows where feto-infant deaths are concentrated, not why. Intrapartum stillbirth and births below 2500 g are the groups in which any further reduction would have to be found.

## 1. Introduction

Each year the world records roughly 1.9 million stillbirths and about 2.3 million newborn deaths, and most of them happen in low- and middle-income settings where the care around labor and the first days of life is thinnest [1,2]. These deaths cluster in a narrow window. A fetus that is healthy at the onset of labor and an infant who survives the first week face very different, and usually much smaller, risks thereafter. That timing is why the two are targeted separately rather than folded into a single figure. Sustainable Development Goal 3.2 sets a neonatal mortality target of 12 or fewer deaths per 1000 live births by 2030 and contains no stillbirth target; stillbirth entered the global framework through the Every Newborn Action Plan, which sets national stillbirth-rate targets alongside neonatal ones [3]. The distinction matters for surveillance because a facility reporting only neonatal outcomes can move toward the SDG indicator while its stillbirth rate stands still [4].

Kazakhstan is a useful place to study this. The country moved to the full World Health Organization definitions of live birth and stillbirth in 2008, which briefly pushed reported mortality upward as very small infants entered the count, and then invested in regionalized perinatal care and new perinatal centers [5,6]. National infant mortality has fallen steadily since, into the single digits per 1000 live births [6,7,8]. What national figures cannot show is where, inside a given hospital, the remaining deaths sit—by gestational timing, by age at death and by birthweight. That is precisely the question a maternity center needs answered before it can decide what to fix.

The BABIES matrix was built for that question. Developed within the Perinatal Periods of Risk framework, it sorts every fetal and infant death into a grid defined by birthweight on one axis and age at death on the other, so that a small number of cells map onto distinct opportunities for prevention: maternal health and antenatal care for the smallest infants, intrapartum care for deaths around birth, and newborn care for deaths in the first weeks [9,10]. The tool is deliberately low-tech, which is why it has been used to drive quality improvement in resource-limited maternity units elsewhere [11]. Despite that, published long-run BABIES analyses from Central Asia are essentially absent.

We had 11 consecutive years of registry data from one tertiary center, together with a birthweight-stratified BABIES matrix. Our aims were straightforward: to describe the trend in stillbirth, neonatal, infant and perinatal mortality from 2015 to 2025; to separate the antenatal, intrapartum, early and late neonatal components; and to use the matrix to ask whether any decline came from fewer small births occurring or from lower composite feto-infant mortality within the birthweight groups themselves. The analysis is ecological throughout, and we treat it as a description of what happened rather than as evidence for why.

## 2. Materials and Methods

### 2.1. Study Design and Setting

This was a retrospective, single-center, ecological time-series analysis of aggregated annual data from one tertiary maternity center in Kazakhstan covering 1 January 2015 through 31 December 2025. The center provides obstetric and neonatal care, including referral-level services, so its case mix is weighted toward higher-risk pregnancies. No patient-level records were used; the unit of analysis was the facility-year. The 2025 reporting year is complete. Counts were extracted from the closed annual registry after the 2025 reporting cycle had been finalized and audited, so every 2025 value in this manuscript is a final observed count, not a projection, an extrapolation or a provisional estimate. Data were extracted on 12 March 2026.

### 2.2. Clinical Context of the Study Period

An aggregated annual series cannot identify what changed clinically, so we set out the policy and service context in which these eleven years sit. Kazakhstan adopted the WHO definitions of live birth and stillbirth in 2008, which raised recorded mortality for several years as very small infants entered the count, and then pursued regionalization of perinatal care with a three-level referral structure and new perinatal centers [5,6,7]. A confidential audit of perinatal mortality was introduced nationally in 2016 with technical support from UNICEF, although uptake across maternity hospitals has been uneven; national perinatal mortality fell from 22.7 per 1000 in 2008 to roughly 9.1 per 1000 in 2023 [12]. The study center operates at the third level of that structure and receives referrals accordingly.

### 2.3. Data Sources

Two sources were combined. The first was the institutional annual registry of deliveries, total births, live births, stillbirths (separated into antenatal and intrapartum), and deaths through the first year of life, split into early neonatal (0–6 days), late neonatal (7–27 days) and postneonatal (28 days to <1 year) periods (Table S1). The second was a BABIES matrix that cross-classified antenatal stillbirths, intrapartum stillbirths and deaths at 0–6 and 7–27 days by four birthweight categories—500–999 g, 1000–1499 g, 1500–2499 g and ≥2500 g (Table S2). Deaths occurring after discharge were not identified from the maternity record alone. Infants born at the center are registered in the national electronic health information system and in the civil registration records, and deaths before the first birthday are notified back to the facility of birth through the territorial health department. That notification route is how late neonatal and postneonatal deaths enter the institutional annual registry. Two consequences follow. Deaths among infants whose families moved outside the catchment during the first year of life may be missed, and any change in the completeness of notification over eleven years would appear in these data as a change in postneonatal mortality. We therefore treat the postneonatal estimates as the least secure of the outcomes reported here, and we note in Section 4.5 that the facility infant mortality rate is not comparable with a population infant mortality rate for the same reason. The compilation route itself should be stated since it determines where errors can enter. Delivery-room and neonatal records are entered into ward logs; the medical statistics office aggregates those logs into the annual institutional report, which is the source for every count in this paper; and the birthweight-stratified BABIES worksheet is prepared separately from the same logs. The worksheet is therefore a parallel product rather than a derivative of the annual report, which is why the two can disagree and why comparing them is a meaningful check rather than a tautology.

### 2.4. Definitions

Stillbirth, perinatal, antenatal and intrapartum rates used total births as the denominator. Early neonatal, late neonatal, neonatal, postneonatal and infant rates used live births. Perinatal mortality was defined as stillbirths plus deaths at 0–6 days per 1000 total births. Low birthweight (LBW) meant <2500 g and very low birthweight (VLBW) <1500 g. Stillbirth was defined primarily by gestational age, with birthweight applied only when gestational age could not be established. A fetal death was registered as a stillbirth at 22 completed weeks of gestation or later; where gestational age was unknown, a birthweight of 500 g or more, or a crown-heel length of 25 cm or more, was used instead. These are the WHO criteria adopted in Kazakhstan in 2008 [5,6]. We understand these criteria to have been applied unchanged across 2015–2025. Antenatal stillbirth meant fetal death before the onset of labor, and intrapartum stillbirth meant fetal death after labor had begun, as recorded by the attending team at delivery. Because the lower bound of the BABIES matrix (500 g) coincides with the lower bound of the registration criteria, no registered death falls outside the matrix. The weight-specific outcome is a composite by design rather than by convenience, and the reason is worth stating. The BABIES matrix and the perinatal-periods-of-risk framework it belongs to are built on age at death from fetal death through day 27, cross-classified by birthweight, with total births in the band as the denominator [9,10,11]. Keeping that structure preserves comparability with published BABIES analyses and keeps stillbirths inside the accounting, which is the point of the tool: a facility that shifts deaths from the neonatal side to the fetal side has not improved, and a neonatal rate alone would not show that. Splitting the composite into its four parts at this volume would also produce cells with single-figure counts in the smallest bands, where the annual numbers run from zero to a handful. The cost is that the composite cannot be read as postnatal survival, and we say so wherever it appears. The 500 g bound is the registration threshold rather than a matrix truncation. In each of the nine internally consistent matrix years, the four birthweight denominators summed exactly to the registry total for births, which they could not do if births below 500 g existed and were being dropped. Infant mortality in this study is a period measure and not a birth-cohort measure. For each calendar year, the numerator is the number of deaths under one year of age recorded in that year among infants born at the center, and the denominator is live births at the center in the same year. No follow-up into the subsequent year is involved, so the 2025 figure is complete on its own terms. It is not the probability that the 2025 birth cohort dies before its first birthday and should not be read as one. The same applies to the postneonatal component. The matrix outcome for each birthweight cell was a composite of antenatal and intrapartum stillbirths plus deaths at 0–27 days—that is, a birth-to-27-day feto-infant mortality indicator. Because the source matrix used total births within each weight band as the denominator and did not provide gestational-age strata, we report this composite rather than a conventional live-birth neonatal rate for the weight-specific analyses.

### 2.5. Data Quality and Reconciliation

Birthweight denominators summed correctly to annual total births in every year. Two problems required decisions, both made before the outcome analyses and documented in Supplementary Materials. First, the weight-specific mortality cells for 2017 and 2018 did not reconcile with the annual registry; these two years were therefore excluded from all birthweight-specific mortality, standardization and decomposition analyses, while their annual aggregate outcomes and birthweight denominators were retained. Second, the 2022 early-neonatal total in the matrix header was inconsistent with the sum of its four weight rows; we used the reconciled value of 34 deaths, which matched the annual registry. Nine of the eleven years were internally consistent and formed the basis for the matrix analyses. The nature of the 2017 and 2018 problem determines how much weight the exclusion should carry, so it is worth stating plainly. The annual registry was internally consistent in both years: antenatal plus intrapartum stillbirths equaled the reported stillbirth total, and early plus late plus postneonatal deaths equaled the reported infant total. What failed was the weight-stratified worksheet. For 2017, the matrix cells summed to 14 deaths against 109 in the registry; for 2018, they summed to 277 against 96. Neither is a small discrepancy that could be adjudicated. Because the fault sits in the weight stratification and not in the annual counts, 2017 and 2018 remain usable for every aggregate analysis and are excluded only where birthweight strata are required. In each of the other nine years, all four matrix components matched the registry exactly (Table S3). Three checks were applied to those nine years rather than one. Each of the four death components matched the annual registry exactly. The four birthweight denominators summed exactly to the registry total for births in every year. And the row-wise and column-wise totals inside each annual matrix agreed with each other. We are explicit about what this does not establish: agreement of margins and components does not prove that an individual death was allocated to the correct birthweight band, and that could only be verified against the original delivery records, which was outside the scope of this analysis. The birthweight-specific results should be read with that residual uncertainty in mind. What the two failed years imply for the other nine is a fair question, and we would rather answer it than leave it open. Two things can be said. The failures were confined to the worksheet and did not appear in the annual report, which was internally consistent in those years, so the evidence points to the worksheet as the weaker of the two documents rather than to a general failure of record-keeping. And the checks that caught 2017 and 2018 were applied identically to every other year and passed in all of them, which means the detection procedure works and that errors of comparable size would not have gone unnoticed. What cannot be excluded is a smaller error that keeps the margins intact, for instance, a death assigned to the wrong weight band. We regard that as the residual risk in the birthweight-specific results and have not tried to argue it away. The 2022 discrepancy was resolved on three independent grounds rather than by preference. The matrix header cell for early neonatal deaths read 63, while the four birthweight rows summed to 34. The row-wise totals of the same matrix sum to 157, which is the grand total printed in the matrix and is reproduced only if early neonatal deaths equal 34; with 63, the column totals would give 186. The annual registry also records 34. The annual registry was treated as authoritative throughout, with the weight rows as corroboration, and the header cell was taken as a transcription error.

### 2.6. Statistical Analysis

Mortality rates were reported with exact Poisson 95% confidence intervals. The 2015-to-2025 change for each outcome was summarized as a rate ratio with a log-scale confidence interval and a Fisher exact test. Annual trends were estimated with Poisson generalized linear models using the logarithm of the relevant birth denominator as an offset and heteroscedasticity-robust standard errors, reported as incidence rate ratios (IRRs) per calendar year. Kendall’s tau served as a nonparametric trend check, and the chi-square test assessed between-year heterogeneity. Overdispersion was monitored with the Pearson chi-square divided by degrees of freedom. Annual trends in low- and very-low-birthweight prevalence were estimated separately by logistic regression with calendar year entered as a continuous predictor and total births as the denominator, and are reported as an odds ratio per calendar year. Poisson models were fitted as generalized linear models with a log link and the logarithm of the relevant birth denominator as an offset, and were reported with Huber-White (HC0) sandwich standard errors. No small-sample correction was applied. With eleven observations, the sandwich estimator is anti-conservative, which is one reason the quasi-Poisson and negative binomial refits described below are reported alongside it; where the three disagree, the wider interval should be preferred. Eleven annual observations leave little room to diagnose the variance function, so each Poisson trend model was refitted in two further ways. The first was a quasi-Poisson model, in which the coefficients are unchanged but the standard errors are scaled by the square root of the estimated dispersion. The second was a negative binomial model, with the dispersion parameter estimated by maximum likelihood and tested against the Poisson by a likelihood-ratio test on the parameter boundary [13]. All three sets of estimates are reported together in Table 1, and the segmented model described below was also refitted as a negative binomial.

**Table 1:** Poisson, quasi-Poisson and negative binomial estimates of the annual trend, 2015–2025. IRR = incidence rate ratio per calendar year. Dispersion is the Pearson chi-square divided by degrees of freedom from the Poisson fit. The likelihood-ratio (LR) test compares the negative binomial against the Poisson on the parameter boundary; a small *p*-value indicates extra-Poisson variation.

Outcome	Dispersion	Poisson IRR (95% CI)	Quasi-Poisson IRR (95% CI)	Negative Binomial IRR (95% CI)	LR Test *p*
Stillbirth	2.85	0.973 (0.943–1.004)	0.973 (0.940–1.008)	0.972 (0.942–1.003)	0.012
Antenatal stillbirth	1.45	0.961 (0.935–0.988)	0.961 (0.935–0.987)	0.961 (0.938–0.984)	0.407
Intrapartum stillbirth	2.98	1.037 (0.972–1.107)	1.037 (0.951–1.131)	1.044 (0.968–1.126)	0.008
Early neonatal	1.56	0.945 (0.911–0.981)	0.945 (0.898–0.995)	0.945 (0.902–0.989)	0.283
Late neonatal	1.33	0.878 (0.846–0.912)	0.878 (0.828–0.932)	0.878 (0.835–0.925)	0.494
Neonatal	1.31	0.918 (0.896–0.940)	0.918 (0.885–0.952)	0.918 (0.888–0.949)	0.447
Postneonatal	0.99	0.914 (0.868–0.962)	0.914 (0.858–0.973)	0.914 (0.858–0.973)	1.000
Infant	1.30	0.917 (0.894–0.941)	0.917 (0.888–0.947)	0.917 (0.891–0.943)	1.000
Perinatal	3.10	0.968 (0.941–0.995)	0.968 (0.937–0.999)	0.966 (0.939–0.994)	0.006

Three descriptive calendar periods (2015–2019, 2020–2022, 2023–2025) were summarized as pooled rates; they are labels of convenience and are not intervention groups. An exploratory segmented Poisson model with level- and slope-change terms was fitted [14]. The break point was fixed at 2020 before any model was estimated, and it was chosen for a reason external to these data: the WHO declaration of the COVID-19 pandemic in March 2020 and the reorganization of maternity services in Kazakhstan that followed it. We did not search across candidate years. With eleven points, a data-driven break would be selected largely on noise, and the resulting *p*-value would not carry its nominal meaning. As a check on how much the prespecified choice matters, the same model was refitted at 2019 and at 2021, and the three fits are compared rather than the best-fitting year being selected. The model is underpowered whichever break is used, and we report it as a description. Special-cause variation was screened with denominator-adjusted p-charts (funnel limits) using the pooled eleven-year rate as the center line and limits of 3.09 standard errors, which widen or narrow each year with the size of the birth denominator [15]. Charts were constructed for stillbirth; antenatal stillbirth; intrapartum stillbirth; and perinatal, neonatal and infant mortality. Following peer review, these charts have been moved to Table S7. Two limitations make them a poor companion to the trend models. A center line drawn from the pooled eleven-year rate sits above the later years and below the earlier ones whenever a secular trend is present, so late points are flagged as low and early points as high partly by construction rather than because the process shifted. And with annual counts this small, the limits are wide, so the absence of a signal carries little information about whether a rate is stable.

The BABIES analyses covered the birthweight distribution, cell-specific composite mortality with pooled rate ratios, direct standardization to the pooled birthweight distribution, and a Kitagawa decomposition separating the 2015-to-2025 change into a within-weight mortality component and a birthweight-composition component. Spearman rank correlations among annual indicators were corrected for multiple comparisons with the Benjamini–Hochberg false-discovery-rate procedure [16]. The decomposition follows Kitagawa [17], with the within-weight component evaluated at the mean of the two birthweight distributions and the composition component at the mean of the two sets of band-specific rates. Because a decomposition anchored on two single years is sensitive to year-to-year variation in the smallest bands, the analysis was repeated comparing pooled early years (2015, 2016 and 2019) with pooled late years (2023 to 2025) using only internally consistent matrix years. Following peer review, the analysis was simplified. The segmented regression and the correlation matrix have been moved out of the main text to Tables S5 and S6. With eleven facility-years, neither adds much, and both are retained in the Supplement for transparency rather than deleted. Analyses were carried out in Python 3.12.3 using NumPy 2.4.4, pandas 3.0.2, SciPy 1.17.1 and statsmodels 0.15.0. All tests were two-sided with α = 0.05, and we treated effect sizes and intervals as more informative than *p*-values given the small number of yearly observations. The study was not prospectively registered, and no analysis plan was deposited before the work began, so we state explicitly which analyses were fixed in advance and which were not. Specified before the data were examined: annual rates with exact Poisson intervals; the 2015 versus 2025 rate ratios; the Poisson trend models; the birthweight-specific composite rates; direct standardization; the Kitagawa decomposition; and the segmented model with its 2020 break. Specified and run in the original analysis but omitted from the first submission: the process-control charts, whose results were added at revision and then moved to the Supplement. Added afterwards: the three-period summary, the correlation matrix, and, in response to peer review, the quasi-Poisson and negative binomial refits, the alternative break years and the pooled-period decomposition. The post hoc analyses are presented as sensitivity and description, and no primary conclusion rests on any of them.

### 2.7. Ethics

The study used aggregated, de-identified institutional statistics with no individual patient data. It was conducted in accordance with the Declaration of Helsinki; institutional review board review and the requirement for informed consent are addressed in the statements at the end of the article. The approval date (16 July 2026) postdates the 2015–2025 observation window, and we set out why. No data were collected for research purposes during the observation window. The records analyzed here are routine institutional statistics that were compiled for administrative reporting and already existed in aggregated, de-identified form when the study was conceived. Ethics review was sought at the point at which secondary analysis of those existing aggregates was planned, which is the normal sequence for retrospective work of this kind, and the committee waived the requirement for informed consent on the grounds that no individual-level or identifiable data were accessed at any stage.

## 3. Results

### 3.1. Birth Volume and Mortality Overview

Annual births rose over the period, from 8144 total births in 2015 to a peak above 11,500 in 2021 before easing to 9589 in 2025. Against that rising and then plateauing volume, every summary mortality rate was lower at the end than at the start (Table 2, Figure 1). The stillbirth rate moved from 10.07 to 5.42 per 1000 total births, perinatal mortality from 12.77 to 6.99, neonatal mortality from 5.58 to 2.62, and infant mortality from 7.07 to 3.57 per 1000 live births. The paths were not smooth: stillbirth and perinatal rates were noisy through the middle years and peaked around 2019 and 2022 before the sharp fall in 2023–2025.

### 3.2. Composition of Feto-Infant Deaths

Across the full period, antenatal deaths made up 82.9% of all stillbirths and intrapartum deaths the remaining 17.1%. Among the 489 infant deaths, 47.4% occurred in the early neonatal period, 32.1% in the late neonatal period and 20.4% after the neonatal period. Figure 2 shows the yearly counts by component. The visible shrinkage from 2022 onward is driven mostly by fewer neonatal deaths; the antenatal stillbirth band narrows only in the final two years, and the thin intrapartum band stays roughly constant throughout.

### 3.3. Temporal Trends

Poisson trend models (Table 3, Figure 3) showed significant annual declines for antenatal stillbirth and early neonatal, late neonatal, neonatal, postneonatal, infant and perinatal mortality. The steepest was late neonatal mortality (IRR 0.878 per year, 95% CI 0.846–0.912), followed by neonatal (0.918) and infant (0.917) mortality, each close to an 8% reduction per calendar year. Total stillbirth trended downward but did not cross the 0.05 threshold (IRR 0.973, *p* = 0.093), and the reason is visible in its parts: antenatal stillbirth fell (IRR 0.961, *p* = 0.004), while intrapartum stillbirth did not move in any consistent direction (IRR 1.037, *p* = 0.272). Kendall’s tau agreed with the model signs for every outcome, but not always with the model *p*-values, and dispersion was not uniformly acceptable. Perinatal mortality is the clearest instance. The Poisson model gives a significant decline (IRR 0.968, *p* = 0.021), while the Kendall test does not (τ = −0.418, *p* = 0.087), and the dispersion statistic for that outcome is 3.10. We no longer describe dispersion as acceptable. Where a Poisson result rests on a dispersion statistic well above 1, the negative binomial estimate in Table 1 should be taken as primary; for perinatal mortality that estimate is still a decline (IRR 0.966, 95% CI 0.939–0.994) but a less certain one than the Poisson fit implied. Because dispersion above 1 can inflate significance under a Poisson likelihood, every trend model was refitted as quasi-Poisson and as negative binomial (Table 1). The point estimates barely moved. Intervals widened where dispersion was highest, and for three outcomes (total stillbirth, intrapartum stillbirth and perinatal mortality), the likelihood-ratio test supported genuine extra-Poisson variation, so the negative binomial row is the one to read for those three. No substantive conclusion changed: the same seven outcomes declined, and the same two did not. Two rows sat closer to the 0.05 boundary under quasi-Poisson than under the Poisson model with robust errors, early neonatal mortality (*p* = 0.031) and perinatal mortality (*p* = 0.046), and should be read with that in mind. Denominator-adjusted process-control charts were also constructed and are reported in Table S7 rather than here. With a series that already carries a secular trend, a center line taken from the pooled eleven-year rate will flag later years as low and earlier years as high partly by construction, so the charts add little to the trend models. One observation from them is worth carrying forward, with its caveat: no annual intrapartum stillbirth value fell outside its limits, but those limits are wide enough that a moderate real change would not have been detected, so this is a statement about low power rather than evidence that the rate was constant.

**Table 3:** Poisson annual incidence rate ratios (IRRs) with robust 95% confidence intervals, nonparametric trend and between-year heterogeneity, 2015–2025.

Outcome	IRR/Year	95% CI	Poisson *p*	Kendall τ	Trend *p*	Dispersion	Het. *p*
Stillbirth	0.973	0.943–1.004	0.093	−0.309	0.218	2.85	<0.001
Antenatal stillbirth	0.961	0.935–0.988	0.004	−0.564	0.017	1.45	0.008
Intrapartum stillbirth	1.037	0.972–1.107	0.272	0.273	0.283	2.98	0.001
Early neonatal	0.945	0.911–0.981	0.003	−0.491	0.041	1.56	0.025
Late neonatal	0.878	0.846–0.912	<0.001	−0.527	0.026	1.33	<0.001
Neonatal	0.918	0.896–0.940	<0.001	−0.636	0.006	1.31	<0.001
Postneonatal	0.914	0.868–0.962	<0.001	−0.491	0.041	0.99	0.077
Infant	0.917	0.894–0.941	<0.001	−0.600	0.010	1.30	<0.001
Perinatal	0.968	0.941–0.995	0.021	−0.418	0.087	3.10	<0.001

### 3.4. Direct Comparison of 2015 and 2025

Comparing the two endpoints directly (Table 4), infant mortality was roughly halved (rate ratio 0.50, 95% CI 0.33–0.77) and neonatal mortality fell by 53%, driven again by the late neonatal component (rate ratio 0.37, *p* = 0.008). Perinatal mortality dropped 45%. The one component for which no clear change was detected was intrapartum stillbirth: 1.11 per 1000 in 2015 versus 1.04 in 2025 (rate ratio 0.94, 95% CI 0.38–2.32, Fisher *p* = 1.000). An interval of that width is compatible with a substantial fall and with a substantial rise, so the result should be read as an absence of evidence for change rather than as evidence of stability.

### 3.5. Calendar Periods

Pooled by period, neonatal and infant mortality fell across the three windows (infant 6.03, then 4.85, then 3.16 per 1000), while stillbirth and perinatal mortality stayed flat through 2015–2022 and dropped only in 2023–2025. An exploratory segmented model with a break fixed at 2020 was also fitted. Following peer review, it has been moved to Table S5, since eleven annual points cannot support a break-point conclusion. One line of it belongs here: the level shift is significant with robust standard errors but not with model-based or negative binomial errors (*p* = 0.078 in both), and refitting the break at 2019 or 2021 removes it altogether. We draw no inference from it.

### 3.6. Birthweight Distribution

Low-birthweight prevalence fell from 6.57% in 2015 to 5.20% in 2025 (annual odds ratio 0.964, 95% CI 0.951–0.977, *p* < 0.001). Very-low-birthweight prevalence hovered between about 1.3% and 1.9% with no clear linear trend (annual OR 0.990, *p* = 0.309). So, the population reaching delivery contained a modestly declining share of small births over the period.

### 3.7. Birthweight-Specific Mortality

The matrix makes the risk gradient stark (Table 5, Figure 4). Pooled over the nine consistent years, composite feto-infant mortality was 681 per 1000 births at 500–999 g, 265 at 1000–1499 g, 52 at 1500–2499 g and just 3.96 at ≥2500 g. Taken together, births below 2500 g carried a composite rate of 154.9 per 1000 against 3.96 in the ≥2500 g group, a pooled rate ratio of 39.1 (95% CI 34.4–44.5). Put the other way, births below 2500 g were 5.9% of all births but accounted for 71.1% of composite feto-infant deaths (Figure 5).

### 3.8. Standardization and Decomposition

Crude composite BABIES mortality fell by 7.56 per 1000 between 2015 and 2025 The Kitagawa decomposition separates the two contributions (Table 6, Figure 6): the within-weight component was −8.01 per 1000 and the composition component +0.45 per 1000, and the two sum to the observed −7.56. We describe this directly rather than as a percentage. The reduction associated with lower composite feto-infant mortality inside birthweight bands would on its own have been slightly larger than the net decline actually observed, and part of it was offset by an adverse shift in the birthweight distribution. Direct standardization to the pooled birthweight distribution barely changed the picture: the crude and standardized series stay close together across the nine consistent matrix years (Figure 7), so the decline was not simply an artifact of fewer small births. Our earlier statement that about 95% of the improvement came from within-weight change used the absolute magnitudes of the two components without defining the calculation, and it is withdrawn. Because a decomposition anchored on two single years is vulnerable to variation in the smallest bands, we repeated it on pooled periods, comparing 2015, 2016 and 2019 with 2023 to 2025 (Table 7). Composite mortality fell from 15.31 to 9.75 per 1000, a change of −5.56, of which −4.65 was within-weight and −0.91 compositional. The within-weight term keeps its direction and remains the larger contribution. The composition term changes sign: pooled across three years at each end, the birthweight distribution moved slightly in a favorable direction rather than an adverse one. The adverse composition effect seen in the single-year comparison therefore reflects the particular pair of years chosen, since the 500–999 g share ranged from 0.49% to 0.99% across the nine consistent years with no monotonic trend. We regard the pooled-period result as the more stable of the two. An exploratory correlation matrix among the annual indicators was also computed and is given in Table S6. Only structurally dependent pairs survived false-discovery-rate correction, and no interpretive weight is placed on it anywhere in this paper.

**Figure 6:**
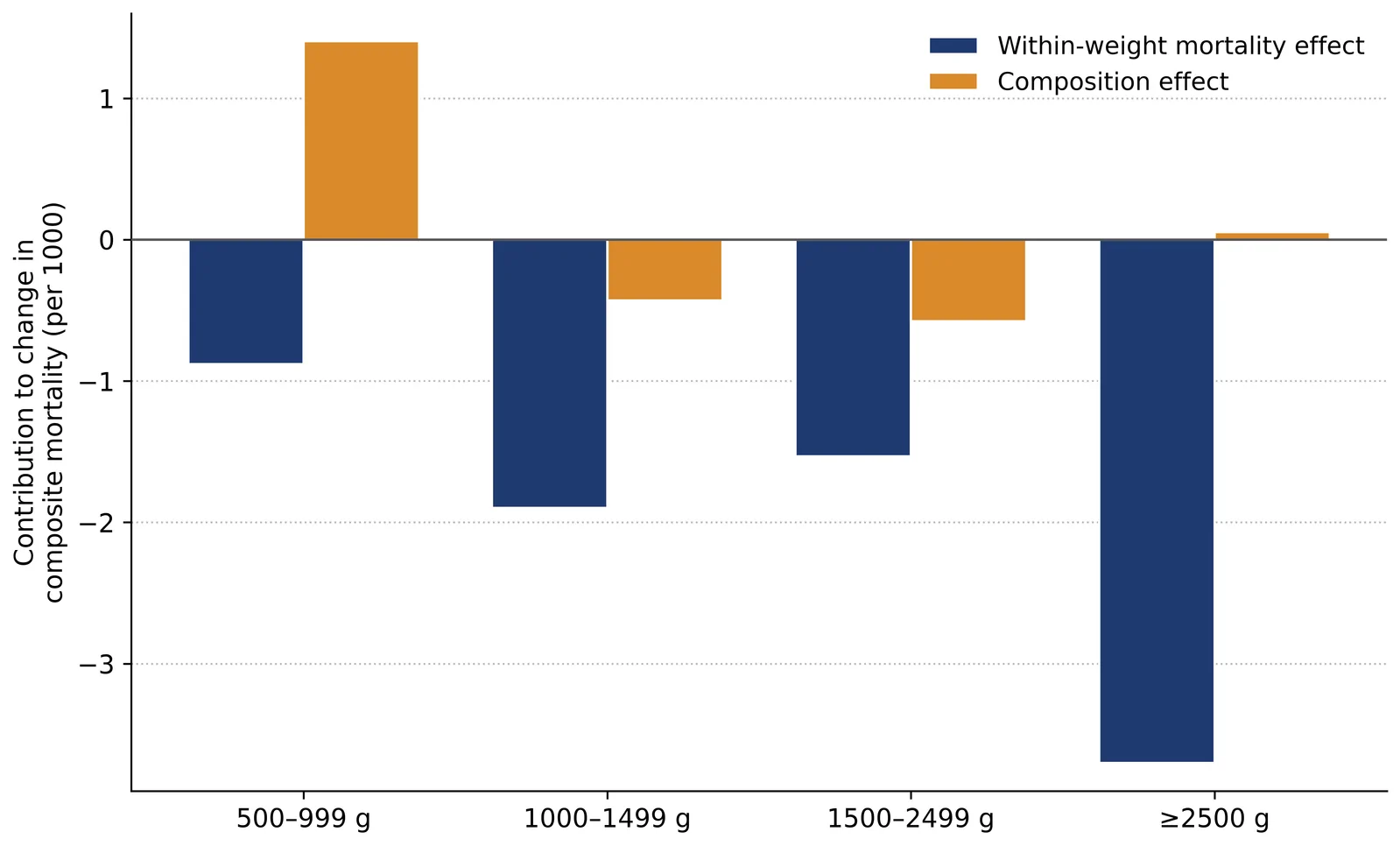
Kitagawa decomposition by birthweight category. Within-weight effects (navy) reflect changing mortality inside each band; composition effects (amber) reflect the changing share of births in each band.

**Figure 7:**
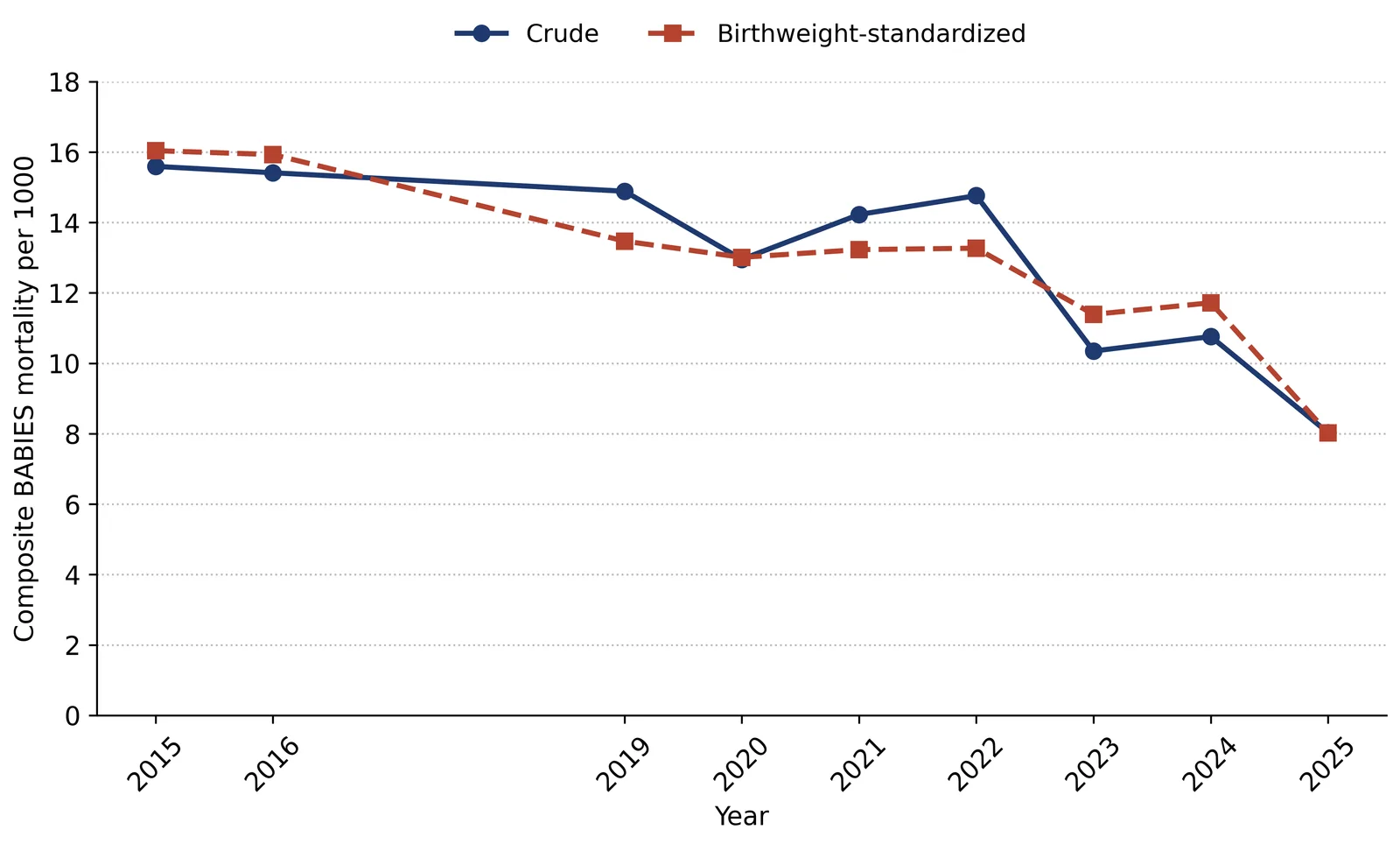
Crude and directly birthweight-standardized composite feto-infant mortality across the nine consistent matrix years.

## 4. Discussion

### 4.1. What the Data Show

We separate observation from explanation in what follows because an aggregated annual series supports the first and not the second.

Four things are observable in these data. Infant and neonatal mortality at this center roughly halved between 2015 and 2025, and the decline held under every model we fitted. Within the neonatal period, the late component fell fastest. Antenatal stillbirth fell, while intrapartum stillbirth did not, and its year-to-year variation stayed inside process-control limits throughout the eleven years. The birthweight decomposition placed the fall in composite feto-infant mortality in the within-weight term rather than the composition term in both the single-year and the pooled-period versions. Composite mortality dropped inside weight bands, while the distribution of birthweight contributed little in either direction.

### 4.2. Possible Explanations, and What Would Be Needed to Test Them

None of that tells us why. The temptation is to read a falling late neonatal rate as evidence that newborn care and follow-up improved. It is a reasonable hypothesis, and we state it as one, but we have no measurements of care to put behind it. Staffing, cot capacity, protocol adoption dates, equipment, training and case severity were not collected, and none of them can be recovered from annual counts. The same downward series would be produced by a shift in who was referred to the center, by a change in how deaths after discharge are notified back to the facility, or by improvement confined to a single unit that annual totals cannot resolve.

A national confidential audit of perinatal deaths in Kazakhstan classified most reviewed cases as involving suboptimal care [12], which indicates that room for improvement existed nationally during our study period. That finding is national and cannot be transferred to this center. Testing any of the explanations above requires patient-level records linked to a documented timeline of practice change, and that is the study we would now undertake.

### 4.3. Intrapartum Stillbirth

Intrapartum stillbirth is the component that did not move, and we want to be careful about how much weight it carries. The 2025 rate (1.04 per 1000 total births) was indistinguishable from the 2015 rate (1.11), with a rate ratio of 0.94 and an interval running from 0.38 to 2.32. An interval that wide is compatible with a substantial fall and with a substantial rise. It does not establish that the rate was constant. What it does establish is that no decline comparable to the antenatal one was detected. We would put it no more strongly than that. The process-control charts in Table S7 flagged no intrapartum year as unusual, but their limits are wide enough that a moderate real change would have passed unnoticed, so that result adds nothing to the confidence interval and we do not treat it as corroboration.

Intrapartum death is generally the stillbirth category most sensitive to monitoring in labor and to the timeliness of intervention [2,4], which is why a flat rate alongside a falling antenatal rate is worth examining. We frame this as a question for audit and not as a finding about labor-ward quality. Without individual records, we cannot separate deaths among previously well term infants, where prevention may have been possible, from deaths among extremely preterm or anomalous fetuses, where it may not. Annual counts here range from 7 to 29, so a case-by-case review is feasible and would settle in a few months what an ecological series cannot settle at all.

### 4.4. Birthweight and the Matrix

The matrix did the job it was designed for. Infants under 2500 g were 5.9% of births and 71.1% of composite deaths, a pooled rate ratio of 39. That gradient is not a discovery, but it converts into an allocation of attention that annual totals do not provide. One caution belongs with it. The weight-specific outcome is a composite of stillbirths and deaths through day 27, calculated on total births within each weight band. It is not a neonatal mortality rate and should not be compared with published neonatal rates. The matrix also has no gestational-age axis, so the group below 2500 g mixes births at 24 weeks with growth-restricted births near term. Those groups differ in cause of death and in prevention route; low birthweight and preterm birth overlap without coinciding, and global burden estimates are produced separately for each [18,19]. Pooling them limits how far the finding can be taken.

The composition term is the part of the decomposition we treat most cautiously. In the single-year comparison, it was slightly adverse; pooled across three years at each end of the series, it was slightly favorable. That reversal comes from the 500–999 g band, which holds between 42 and 105 births a year and whose share moved between 0.49% and 0.99% without a trend. We had previously read a rising share of extremely small infants as a possible sign of maturing neonatal services. That reading does not survive the pooled analysis, and it was not testable with aggregate data in any case. What can be said is narrower: the birthweight distribution changed very little across eleven years and contributed little in either direction, so the decline in composite mortality has to be located inside the weight bands.

### 4.5. Comparison with National Figures

Our 2025 infant mortality of 3.57 per 1000 live births sits well below the national figure of 6.80 per 1000 reported for 2024 [20], and the gap deserves comment rather than celebration. The two numbers are not the same measurement. A national infant mortality rate counts deaths among all infants in a population, wherever they were born and wherever they die. A facility figure counts deaths among infants born at one hospital and depends on that hospital learning about deaths that occur after discharge, through the notification route described in Section 2.3. Incomplete notification would push the facility figure down. A tertiary referral case mix would push it up. We can quantify neither, so we present the facility rate as an internal tracking measure and not as an estimate of population risk. The same caution applies to any comparison between this center and others, and it is one reason we make no claim that these results generalize beyond the institution that produced them.

### 4.6. Strengths and Limitations

The main strengths are the eleven-year span, the internal consistency checks that let us quarantine the corrupted 2017–2018 mortality cells rather than propagate them, and the use of standardization and decomposition to distinguish composition from within-group change. Reporting a long-run facility BABIES analysis from Central Asia also fills a genuine gap.

The limitations are those of any ecological study. We had annual counts, not individual records, so we could not adjust for maternal age, parity, gestational age, referral status, congenital anomalies, mode of delivery or clinical severity, and we cannot attribute any change to a specific intervention. Registration practices may have shifted over eleven years, and we assumed consistent ascertainment. The matrix begins at 500 g and offers no gestational-age strata, so its weight-specific outcome is a birth-to-27-day composite rather than a conventional neonatal rate. The 2017 and 2018 weight-specific cells were unusable, which breaks the matrix time series and reduces power. Finally, rare outcomes such as intrapartum and postneonatal death rest on small annual numbers, and the correlation and segmented analyses are underpowered with only eleven points. Three further limitations should be read alongside those. The study was not prospectively registered, and the analyses added during revision were not prespecified, as set out in Section 2.6. Ascertainment of deaths after discharge depends on a notification route we could not audit, which makes postneonatal mortality the least secure outcome and the facility infant mortality rate non-comparable with population rates. And the manuscript contains no measurement of clinical practice at the center, so every explanation offered in Section 4.2 is a hypothesis rather than a finding. Two further points belong here. The matrix was validated against the annual registry at the level of margins and components, which does not establish that individual deaths were allocated to the correct birthweight band; verifying that would require the original delivery records. And infant mortality here is a period measure rather than a birth-cohort measure, so it should not be compared with cohort probabilities of death before the first birthday.

## 5. Conclusions

Between 2015 and 2025, stillbirth and neonatal, infant and perinatal mortality at this tertiary maternity center all ended the period lower than they began. Separately, and for one outcome only, the birthweight decomposition of composite feto-infant mortality places that particular change within the weight bands rather than in a shift of the birthweight distribution. The decomposition applies to the composite outcome alone and says nothing about the other mortality measures listed above; the temporal trend in late neonatal mortality is a separate result, and we do not combine the two. These are descriptions of what the institutional registry recorded. They are not evidence about what produced the change, and we make no claim about which clinical practices were responsible.

Two features of the series indicate where a patient-level study would be most useful. No clear decline in intrapartum stillbirth was detected across eleven years, and the data cannot exclude a moderate change in either direction. Births below 2500 g remained a small share of all births and accounted for the majority of composite feto-infant deaths. A case-by-case review of intrapartum deaths and a chart-based analysis of outcomes below 2500 g are both feasible at this volume and would answer questions that aggregated annual counts cannot.

## Figures and Tables

**Figure 1:**
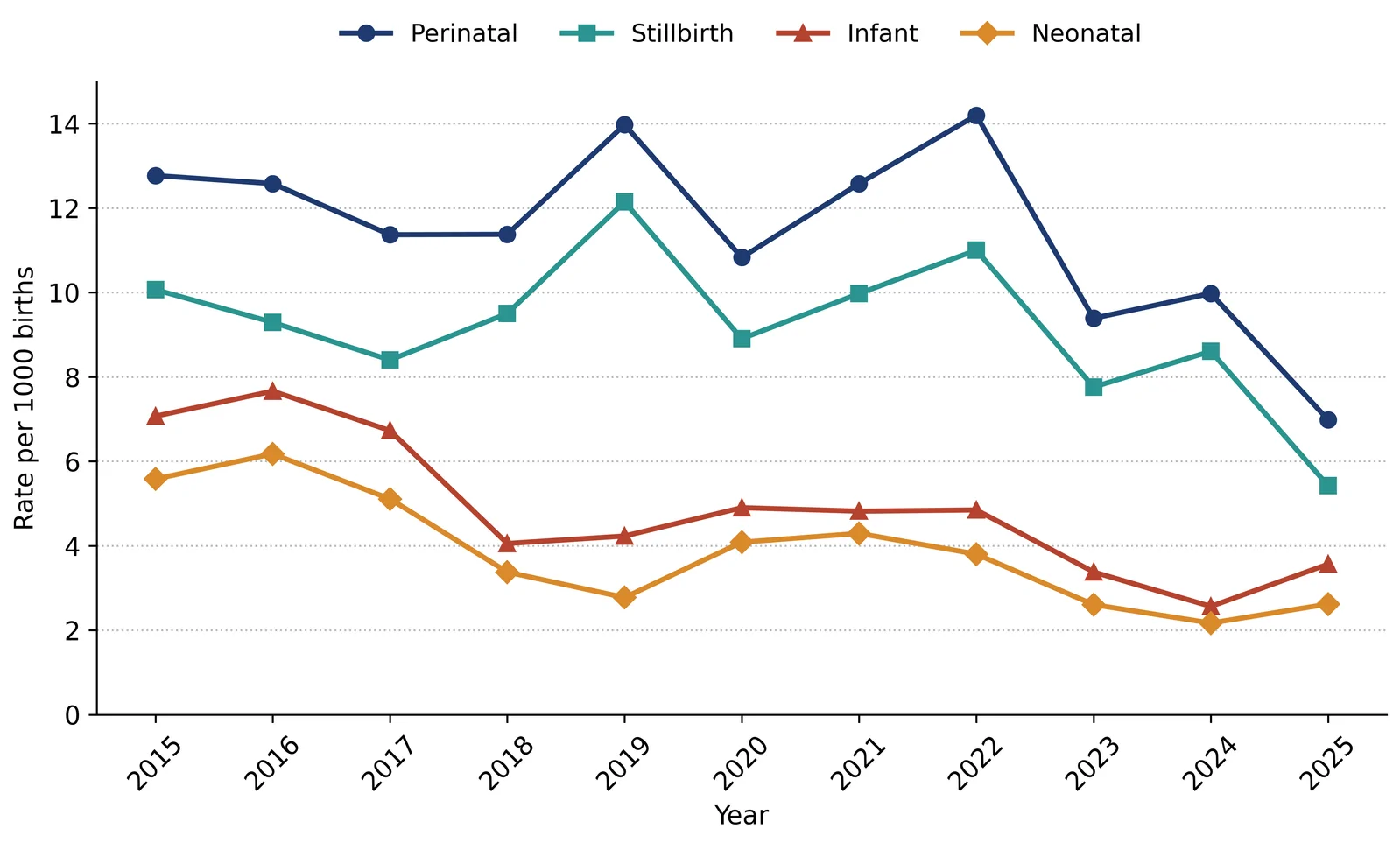
Annual stillbirth, perinatal, neonatal and infant mortality rates, 2015–2025. Stillbirth and perinatal rates are per 1000 total births; neonatal and infant rates are per 1000 live births.

**Figure 2:**
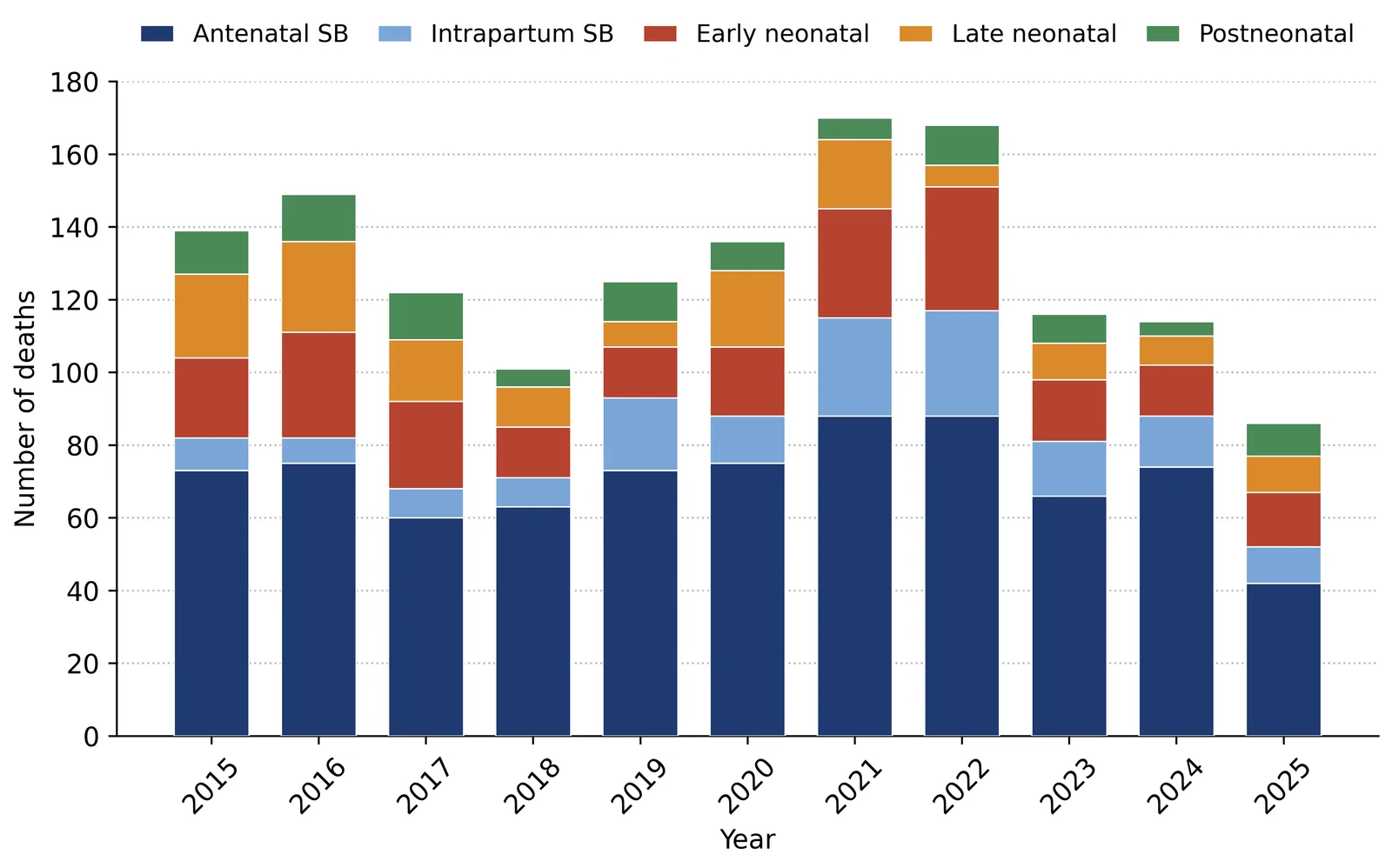
Annual counts of antenatal stillbirths; intrapartum stillbirths; and early neonatal, late neonatal and postneonatal deaths, 2015–2025.

**Figure 3:**
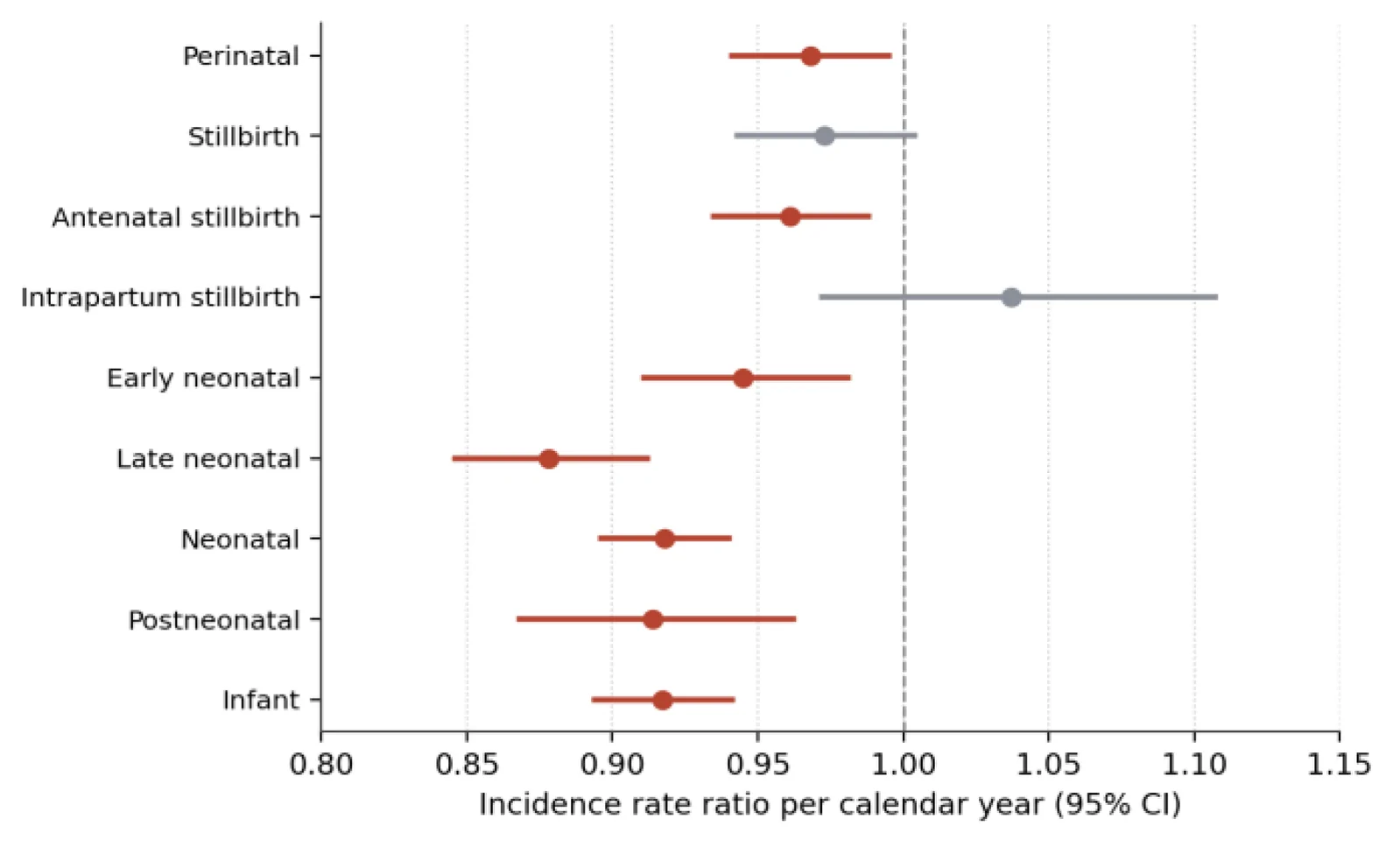
Annual incidence rate ratios from Poisson trend models with 95% confidence intervals. Red intervals lie entirely below 1 (declining); grey intervals cross 1. The vertical dashed line marks no annual change.

**Figure 4:**
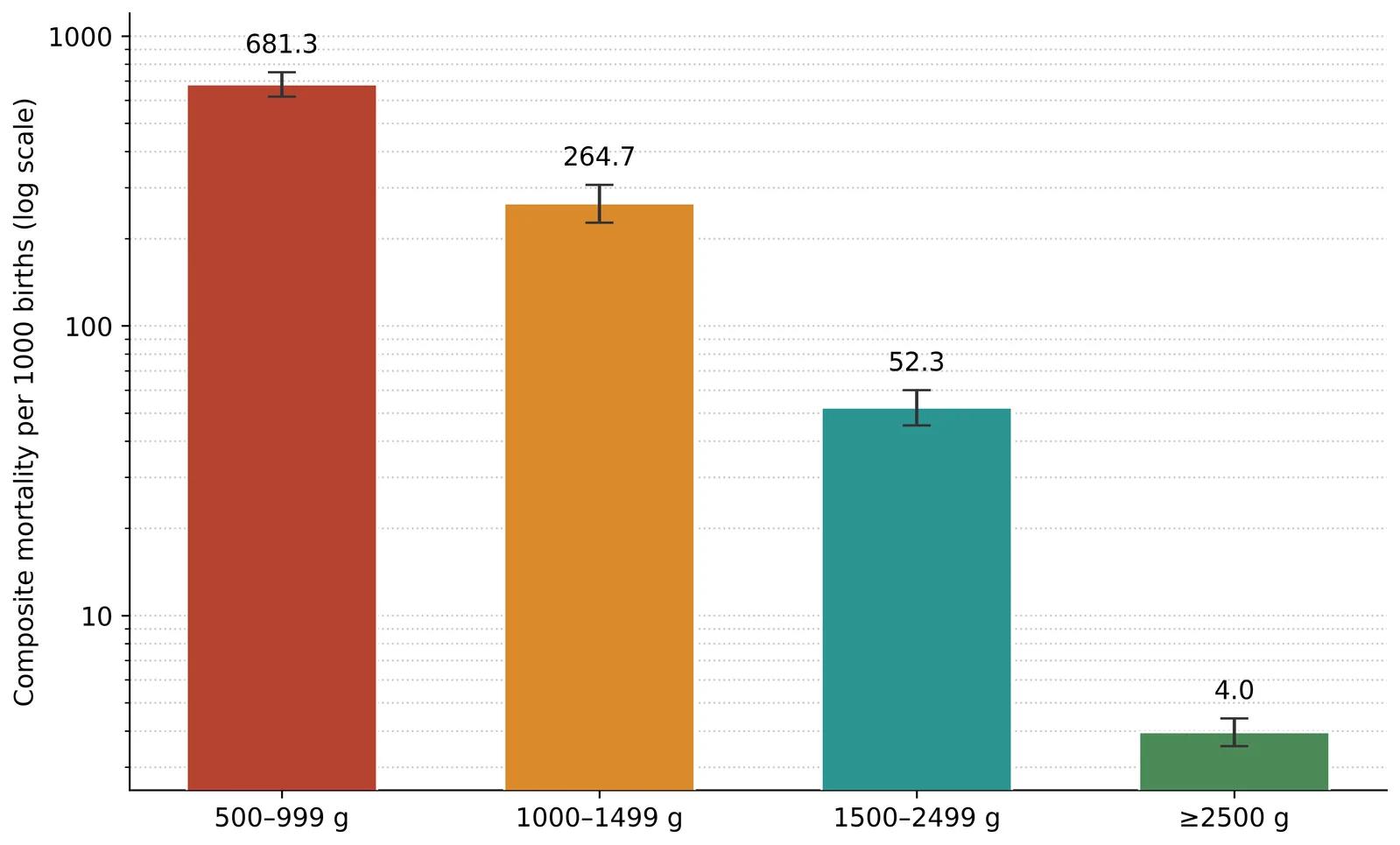
Pooled birthweight-specific composite feto-infant mortality per 1000 births (logarithmic scale) with exact 95% confidence intervals.

**Figure 5:**
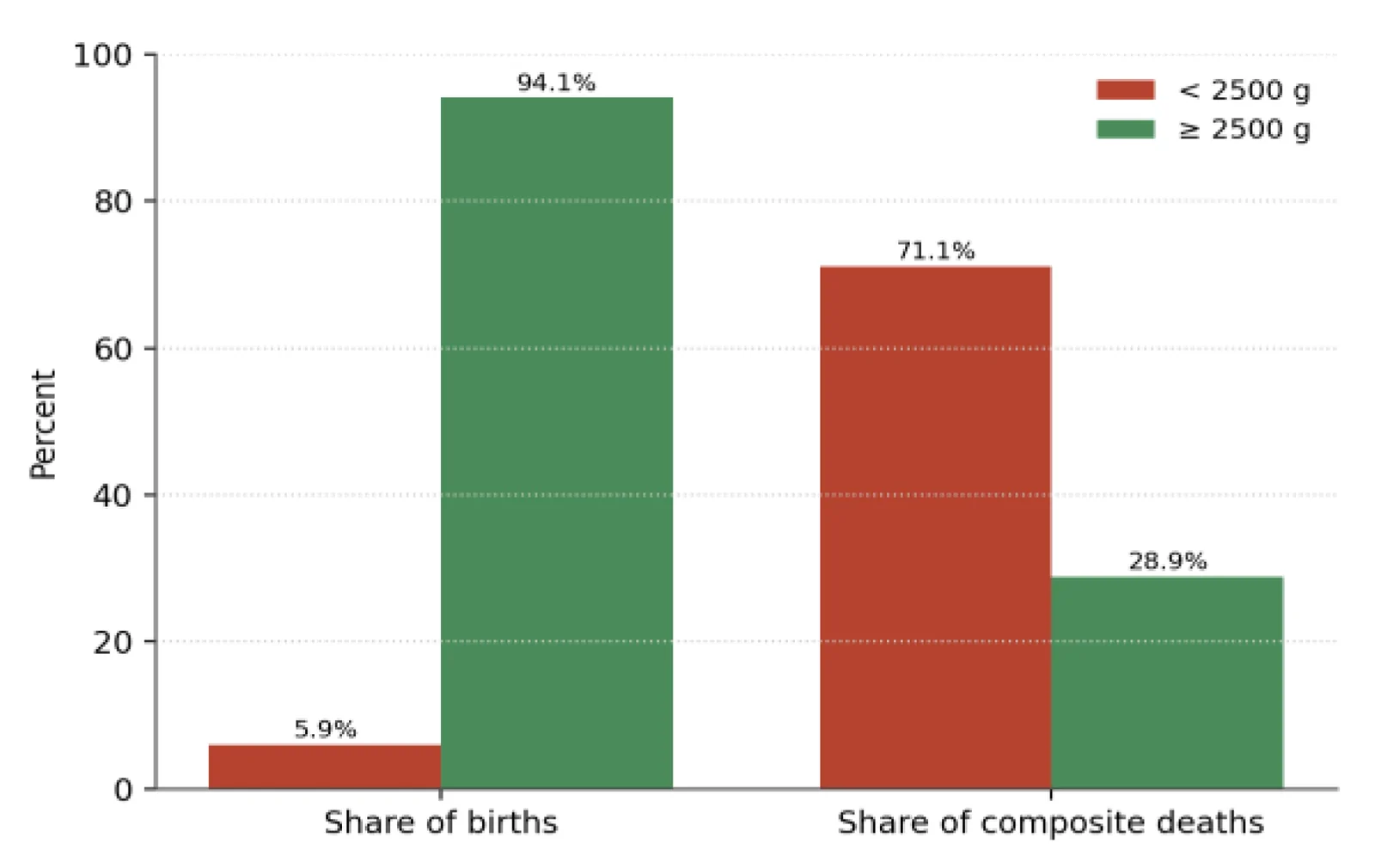
Share of births and share of composite feto-infant deaths for births below and at or above 2500 g, pooled over consistent matrix years.

**Table 2:** Annual births and mortality rates per 1000 with exact Poisson 95% confidence intervals, 2015–2025.

Year	Total Births	Live Births	Stillbirth (95% CI)	Perinatal (95% CI)	Neonatal (95% CI)	Infant (95% CI)
2015	8144	8062	10.07 (8.01–12.50)	12.77 (10.43–15.47)	5.58 (4.07–7.47)	7.07 (5.35–9.16)
2016	8824	8742	9.29 (7.39–11.53)	12.58 (10.35–15.15)	6.18 (4.64–8.06)	7.66 (5.94–9.73)
2017	8093	8025	8.40 (6.52–10.65)	11.37 (9.16–13.94)	5.11 (3.67–6.93)	6.73 (5.06–8.78)
2018	7471	7400	9.50 (7.42–11.99)	11.38 (9.09–14.07)	3.38 (2.19–4.99)	4.05 (2.74–5.79)
2019	7657	7564	12.15 (9.80–14.88)	13.97 (11.45–16.89)	2.78 (1.72–4.24)	4.23 (2.89–5.97)
2020	9881	9793	8.91 (7.14–10.97)	10.83 (8.87–13.09)	4.08 (2.92–5.56)	4.90 (3.61–6.50)
2021	11,528	11,413	9.98 (8.24–11.97)	12.58 (10.61–14.80)	4.29 (3.18–5.68)	4.82 (3.63–6.27)
2022	10,635	10,518	11.00 (9.10–13.18)	14.20 (12.02–16.65)	3.80 (2.72–5.18)	4.85 (3.61–6.38)
2023	10,436	10,355	7.76 (6.16–9.65)	9.39 (7.62–11.44)	2.61 (1.72–3.79)	3.38 (2.35–4.70)
2024	10,223	10,135	8.61 (6.90–10.61)	9.98 (8.14–12.11)	2.17 (1.36–3.29)	2.57 (1.68–3.76)
2025	9589	9537	5.42 (4.05–7.11)	6.99 (5.41–8.87)	2.62 (1.70–3.87)	3.57 (2.47–4.98)

**Table 4:** Rate ratios comparing 2025 with 2015 by outcome.

Outcome	2015	2025	Rate Ratio	95% CI	Rel. Change	Fisher *p*
Stillbirth	10.07	5.42	0.539	0.381–0.762	−46.1%	<0.001
Antenatal stillbirth	8.96	4.38	0.489	0.334–0.714	−51.1%	<0.001
Intrapartum stillbirth	1.11	1.04	0.944	0.383–2.322	−5.6%	1.000
Early neonatal	2.73	1.57	0.576	0.299–1.111	−42.4%	0.101
Late neonatal	2.85	1.05	0.368	0.175–0.772	−63.2%	0.008
Neonatal	5.58	2.62	0.470	0.288–0.766	−53.0%	0.002
Postneonatal	1.49	0.94	0.634	0.267–1.505	−36.6%	0.382
Infant	7.07	3.57	0.504	0.330–0.771	−49.6%	0.001
Perinatal	12.77	6.99	0.547	0.402–0.744	−45.3%	<0.001

**Table 5:** Pooled birthweight-specific composite feto-infant mortality across the nine internally consistent matrix years.

Birthweight	Total Births	Composite Deaths	Rate/1000	95% CI	AN	IP	0–6 Day	7–27 Day
500–999 g	615	419	681.30	617.6–749.8	170	101	92	56
1000–1499 g	665	176	264.66	227.0–306.8	91	12	30	43
1500–2499 g	3866	202	52.25	45.3–60.0	140	13	31	18
≥2500 g	81,771	324	3.96	3.54–4.42	253	18	41	12

**Table 6:** Kitagawa decomposition of the 2015-to-2025 change in composite feto-infant mortality (per 1000 total births).

Birthweight	Share 2015	Share 2025	Rate 2015	Rate 2025	Within-Weight	Composition	Total
500–999 g	0.52%	0.75%	666.67	527.78	−0.880	+1.404	+0.525
1000–1499 g	0.87%	0.73%	422.54	185.71	−1.897	−0.431	−2.328
1500–2499 g	5.18%	3.72%	56.87	22.41	−1.534	−0.578	−2.113
≥2500 g	93.43%	94.80%	5.91	1.98	−3.702	+0.054	−3.648

**Table 7:** Kitagawa decomposition repeated on pooled periods, comparing 2015, 2016 and 2019 with 2023 to 2025. Only internally consistent matrix years are used. The outcome is composite feto-infant mortality (antenatal and intrapartum stillbirth plus death at 0–27 days) per 1000 total births in each birthweight band.

Birthweight Band	Share of Births, Early	Share of Births, Late	Rate/1000, Early	Rate/1000, Late	Within-Weight Component	Composition Component
500–999 g	0.59%	0.65%	703.45	578.68	−0.77	+0.36
1000–1499 g	0.92%	0.70%	323.01	206.57	−0.94	−0.58
1500–2499 g	5.39%	3.84%	62.55	42.20	−1.06	−0.81
≥2500 g	93.10%	94.81%	5.19	3.07	−1.99	+0.07
Total	100%	100%	15.31	9.75	−4.65	−0.91

Observed change −5.56 per 1000 = within-weight (−4.65) + composition (−0.91). Early period: 24,625 births, 377 composite deaths. Late period: 30,248 births, 295 composite deaths. For comparison, the single-year 2015 versus 2025 decomposition in Table 6 gives −7.56 = −8.01 within-weight plus +0.45 composition.

## Data Availability

The aggregated data supporting the reported results are contained within the article and its Supplementary Materials. Further requests can be directed to the corresponding author.

## Supplementary Materials

The following supporting information can be downloaded at: https://www.mdpi.com/article/10.3390/children13101284/s1, Table S1: Annual deliveries, births, stillbirths and infant deaths, 2015–2025; Table S2: BABIES matrix as recorded, by birthweight band and year, 2015–2025; Table S3: Year-by-year reconciliation of the BABIES matrix against the annual registry; Table S4: Birthweight-band denominators and composite feto-infant deaths by year, internally consistent matrix years only; Table S5: Exploratory segmented Poisson model: level shift at the prespecified 2020 break, alternative break years and alternative variance estimators; Table S6: Spearman rank correlations among annual indicators, with Benjamini–Hochberg adjusted q-values (n = 11 annual observations); and Table S7: Denominator-adjusted process-control (funnel) charts, 2015–2025: years falling outside their limits.
