# Supplementary material for: Eleven-Year Trends in Stillbirth, Neonatal and Infant Mortality and Birthweight-Specific Risk: A BABIES Matrix Analysis from a Tertiary Maternity Center in Kazakhstan, 2015–2025
Source: Children (Basel). 2026 Sep 22;13(10):1284. doi: 10.3390/children13101284 (PMC13703296; doi:10.3390/children13101284)
Supplement: Supplementary file 1 [file children-13-01284-s001.zip › children-4519154-supplementary.pdf]

Supplementary Materials

Eleven-Year Trends in Stillbirth, Neonatal and Infant Mortality and Birthweight-Specific Risk: A BABIES Matrix Analysis from a Tertiary Maternity Center in Kazakhstan, 2015–2025

Table S1. Annual deliveries, births, stillbirths and infant deaths, 2015–2025.

AN = antenatal stillbirth; IP = intrapartum stillbirth; EN = early neonatal death (0–6 days); LN = late neonatal death (7–27 days); PN = postneonatal death (28 days to under 1 year). Infant deaths are a period measure: deaths under one year recorded in the calendar year among infants born at the centre. Rates for stillbirth and perinatal mortality use total births as denominator; neonatal, postneonatal and infant rates use live births.

Year	Deliveries	Total Births	Live Births	Stillbirths	AN	IP	EN	LN	PN	Neonatal	Infant	Perinatal	Stillbirth/1,000	Neonatal/1,000	Infant/1,000	Perinatal/1,000
2015	8,059	8,144	8,062	82	73	9	22	23	12	45	57	104	10.07	5.58	7.07	12.77
2016	8,723	8,824	8,742	82	75	7	29	25	13	54	67	111	9.29	6.18	7.66	12.58
2017	7,986	8,093	8,025	68	60	8	24	17	13	41	54	92	8.40	5.11	6.73	11.37
2018	7,373	7,471	7,400	71	63	8	14	11	5	25	30	85	9.50	3.38	4.05	11.38
2019	7,545	7,657	7,564	93	73	20	14	7	11	21	32	107	12.15	2.78	4.23	13.97
2020	9,755	9,881	9,793	88	75	13	19	21	8	40	48	107	8.91	4.08	4.90	10.83
2021	11,393	11,528	11,413	115	88	27	30	19	6	49	55	145	9.98	4.29	4.82	12.58
2022	10,488	10,635	10,518	117	88	29	34	6	11	40	51	151	11.00	3.80	4.85	14.20
2023	10,299	10,436	10,355	81	66	15	17	10	8	27	35	98	7.76	2.61	3.38	9.39
2024	10,075	10,223	10,135	88	74	14	14	8	4	22	26	102	8.61	2.17	2.57	9.98
2025	9,473	9,589	9,537	52	42	10	15	10	9	25	34	67	5.42	2.62	3.57	6.99
Total	101,169	102,481	101,544	937	777	160	232	157	100	389	489	1169	9.14	3.83	4.82	11.41

Table S2. BABIES matrix as recorded, by birthweight band and year, 2015–2025.

Values are reproduced as they appear in the institutional matrix worksheet, including the 2017 and 2018 years that failed reconciliation and were excluded from all birthweight-specific analyses (see Table S3). The 2022 early neonatal figures are the birthweight-row values, which sum to 34 and match the annual registry; the matrix header cell for that year read 63 and was treated as a transcription error.

Year	Birthweight band	Total births	Live births	AN	IP	EN (0–6 d)	LN (7–27 d)	Composite deaths	Composite /1,000
2015	500–999 g	42	25	11	6	5	6	28	666.7
	1000–1499 g	71	60	11	0	8	11	30	422.5
	1500–2499 g	422	406	16	0	4	4	24	56.9
	≥2500 g	7,609	7,571	35	3	5	2	45	5.9
	All bands	8,144	8,062	73	9	22	23	127	15.6
2016	500–999 g	43	32	9	2	14	11	36	837.2
	1000–1499 g	87	73	11	3	4	10	28	321.8
	1500–2499 g	482	458	23	1	5	2	31	64.3
	≥2500 g	8,212	8,179	32	1	6	2	41	5.0
	All bands	8,824	8,742	75	7	29	25	136	15.4
2017 *	500–999 g	54	39	2	2	1	0	5	92.6
	1000–1499 g	74	63	2	0	1	1	4	54.1
	1500–2499 g	416	403	2	0	1	0	3	7.2
	≥2500 g	7,549	7,520	2	0	0	0	2	0.3

	All bands	8,093	8,025	8	2	3	1	14	1.7
2018 *	500–999 g	45	29	22	10	20	17	69	1533.3
	1000–1499 g	94	81	24	3	13	22	62	659.6
	1500–2499 g	415	401	41	1	10	6	58	139.8
	≥2500 g	6,917	6,889	69	4	11	4	88	12.7
	All bands	7,471	7,400	156	18	54	49	277	37.1
2019	500–999 g	60	31	13	16	6	3	38	633.3
	1000–1499 g	68	54	12	2	0	1	15	220.6
	1500–2499 g	423	402	20	1	5	2	28	66.2
	≥2500 g	7,106	7,077	28	1	3	1	33	4.6
	All bands	7,657	7,564	73	20	14	7	114	14.9
2020	500–999 g	69	37	24	8	6	10	48	695.7
	1000–1499 g	72	61	10	1	2	8	21	291.7
	1500–2499 g	464	451	11	2	5	1	19	40.9
	≥2500 g	9,276	9,244	30	2	6	2	40	4.3
	All bands	9,881	9,793	75	13	19	21	128	13.0
2021	500–999 g	99	46	30	23	17	10	80	808.1
	1000–1499 g	78	65	12	1	4	3	20	256.4
	1500–2499 g	511	490	20	1	3	3	27	52.8
	≥2500 g	10,840	10,812	26	2	6	3	37	3.4
	All bands	11,528	11,413	88	27	30	19	164	14.2
2022	500–999 g	105	50	30	25	18	2	75	714.3
	1000–1499 g	76	65	11	0	6	1	18	236.8
	1500–2499 g	403	384	18	1	3	2	24	59.6
	≥2500 g	10,051	10,019	29	3	7	1	40	4.0
	All bands	10,635	10,518	88	29	34	6	157	14.8
2023	500–999 g	64	41	15	8	9	6	38	593.8
	1000–1499 g	74	62	11	1	3	3	18	243.2
	1500–2499 g	367	356	8	3	3	1	15	40.9
	≥2500 g	9,931	9,896	32	3	2	0	37	3.7
	All bands	10,436	10,355	66	15	17	10	108	10.3
2024	500–999 g	61	30	23	8	6	1	38	623.0
	1000–1499 g	69	61	7	1	2	3	13	188.4
	1500–2499 g	437	416	17	4	2	3	26	59.5
	≥2500 g	9,656	9,628	27	1	4	1	33	3.4
	All bands	10,223	10,135	74	14	14	8	110	10.8
2025	500–999 g	72	52	15	5	11	7	38	527.8
	1000–1499 g	70	61	6	3	1	3	13	185.7
	1500–2499 g	357	350	7	0	1	0	8	22.4
	≥2500 g	9,090	9,074	14	2	2	0	18	2.0
	All bands	9,589	9,537	42	10	15	10	77	8.0

** 2017 and 2018 failed reconciliation against the annual registry and are excluded from all birthweight-specific analyses.*

Table S3. Year-by-year reconciliation of the BABIES matrix against the annual registry.

Three checks were applied in each year: each of the four death components against the annual registry; the sum of the four birthweight denominators against the registry total for births; and the internal agreement of row-wise and column-wise totals. Agreement of margins and components does not establish that an individual death was allocated to the correct birthweight band, which would require the original delivery records.

Year	Matrix AN	Registry AN	Matrix IP	Registry IP	Matrix EN	Registry EN	Matrix LN	Registry LN	Matrix deaths	Registry deaths	Deaths check	Denominator check	Decision
2015	73	73	9	9	22	22	23	23	127	127	Match	Match	Included
2016	75	75	7	7	29	29	25	25	136	136	Match	Match	Included
2017	8	60	2	8	3	24	1	17	14	109	Mismatch	Match	Excluded (weight-specific only)
2018	156	63	18	8	54	14	49	11	277	96	Mismatch	Match	Excluded (weight-specific only)
2019	73	73	20	20	14	14	7	7	114	114	Match	Match	Included
2020	75	75	13	13	19	19	21	21	128	128	Match	Match	Included
2021	88	88	27	27	30	30	19	19	164	164	Match	Match	Included
2022	88	88	29	29	34	34	6	6	157	157	Match	Match	Included (EN header 63 corrected to 34)
2023	66	66	15	15	17	17	10	10	108	108	Match	Match	Included
2024	74	74	14	14	14	14	8	8	110	110	Match	Match	Included
2025	42	42	10	10	15	15	10	10	77	77	Match	Match	Included

Table S4. Birthweight-band denominators and composite feto-infant deaths by year, internally consistent matrix years only.

Composite feto-infant mortality is antenatal stillbirth, intrapartum stillbirth and death at 0–27 days, expressed per 1,000 total births in the band. It is not a neonatal mortality rate and should not be compared with published neonatal rates.

Year	Total births	500–999 g births (%)	Deaths (rate)	1000–1499 g births (%)	Deaths (rate)	1500–2499 g births (%)	Deaths (rate)	≥2500 g births (%)	Deaths (rate)
2015	8,144	42 (0.52%)	28 (666.7)	71 (0.87%)	30 (422.5)	422 (5.18%)	24 (56.9)	7,609 (93.43%)	45 (5.9)
2016	8,824	43 (0.49%)	36 (837.2)	87 (0.99%)	28 (321.8)	482 (5.46%)	31 (64.3)	8,212 (93.06%)	41 (5.0)
2019	7,657	60 (0.78%)	38 (633.3)	68 (0.89%)	15 (220.6)	423 (5.52%)	28 (66.2)	7,106 (92.80%)	33 (4.6)
2020	9,881	69 (0.70%)	48 (695.7)	72 (0.73%)	21 (291.7)	464 (4.70%)	19 (40.9)	9,276 (93.88%)	40 (4.3)
2021	11,528	99 (0.86%)	80 (808.1)	78 (0.68%)	20 (256.4)	511 (4.43%)	27 (52.8)	10,840 (94.03%)	37 (3.4)
2022	10,635	105 (0.99%)	75 (714.3)	76 (0.71%)	18 (236.8)	403 (3.79%)	24 (59.6)	10,051 (94.51%)	40 (4.0)
2023	10,436	64 (0.61%)	38 (593.8)	74 (0.71%)	18 (243.2)	367 (3.52%)	15 (40.9)	9,931 (95.16%)	37 (3.7)
2024	10,223	61 (0.60%)	38 (623.0)	69 (0.67%)	13 (188.4)	437 (4.27%)	26 (59.5)	9,656 (94.45%)	33 (3.4)
2025	9,589	72 (0.75%)	38 (527.8)	70 (0.73%)	13 (185.7)	357 (3.72%)	8 (22.4)	9,090 (94.80%)	18 (2.0)
Pooled	86,917	615 (0.71%)	419 (681.3)	665 (0.77%)	176 (264.7)	3,866 (4.45%)	202 (52.3)	81,771 (94.08%)	324 (4.0)

Pooled across the nine consistent years, infants below 2500 g were 5,146 of 86,917 births (5.9%) and 797 of 1,121 composite deaths (71.1%), a rate of 154.9 against 4.0 per 1,000 for infants at or above 2500 g (rate ratio 39.1).

Table S5. Exploratory segmented Poisson model: level shift at the prespecified 2020 break, alternative break years and alternative variance estimators.

The break year was fixed at 2020 before any model was estimated, on grounds external to the data (the March 2020 pandemic declaration and the reorganisation of maternity services that followed). No search across candidate years was performed. This analysis was moved from the main text to the Supplement at peer review: with eleven annual observations a segmented model is underpowered, and the level shift does not survive a change of variance estimator or a change of break year. No conclusion in the paper rests on it.

Outcome	Break year	Level-shift IRR	p, robust (HC0)	p, model-based	Negative binomial IRR (95% CI)	p, negative binomial
Neonatal	2020	1.73	<0.001	0.016	1.73 (1.11–2.71)	0.016
Neonatal	2019	1.18	—	0.494	—	—
Neonatal	2021	1.41	—	0.116	—	—
Infant	2020	1.42	0.010	0.078	1.42 (0.96–2.11)	0.078
Infant	2019	1.09	—	0.699	—	—
Infant	2021	1.27	—	0.256	—	—
Stillbirth	2020	1.11	—	0.487	1.11 (0.79–1.56)	0.548
Perinatal	2020	1.15	—	0.294	1.15 (0.85–1.55)	0.372

Table S6. Spearman rank correlations among annual indicators, with Benjamini–Hochberg adjusted q-values (n = 11 annual observations).

Exploratory and reported for completeness only. The two pairs that survive correction share numerator terms by construction and carry no information about mechanism. With eleven observations all associations are dominated by shared secular trend and none can be read as a patient-level relationship. LBW = low birthweight (<2500 g); VLBW = very low birthweight (<1500 g).

Variable 1	Variable 2	Spearman ϱ	Raw p	FDR q	Interpretation
Neonatal mortality rate	Infant mortality rate	0.964	<0.001	<0.001	Structural
Stillbirth rate	Perinatal mortality rate	0.936	<0.001	<0.001	Structural
Total births	LBW prevalence (%)	−0.800	0.003	0.022	Survives correction
Infant mortality rate	Perinatal mortality rate	0.564	0.071	0.280	Not significant
Neonatal mortality rate	Perinatal mortality rate	0.518	0.102	0.280	Not significant
VLBW prevalence (%)	Perinatal mortality rate	0.500	0.117	0.280	Not significant
LBW prevalence (%)	Perinatal mortality rate	0.482	0.133	0.280	Not significant
LBW prevalence (%)	VLBW prevalence (%)	0.473	0.142	0.280	Not significant
LBW prevalence (%)	Stillbirth rate	0.473	0.142	0.280	Not significant
LBW prevalence (%)	Neonatal mortality rate	0.473	0.142	0.280	Not significant
VLBW prevalence (%)	Stillbirth rate	0.464	0.151	0.280	Not significant
LBW prevalence (%)	Infant mortality rate	0.455	0.160	0.280	Not significant

Table S7. Denominator-adjusted process-control (funnel) charts, 2015–2025: years falling outside their limits.

The centre line is the pooled eleven-year rate and the limits are 3.09 standard errors, recalculated each year from that year's birth denominator. Only years producing a signal are listed. Two limitations should be read with this table. Because the underlying series carries a secular trend, a centre line drawn from the pooled rate lies above the later years and below the earlier ones, so late points are flagged low and early points high partly by construction rather than because the process shifted. And with annual counts of this size the limits are wide, so the absence of a signal carries little information about whether a rate was stable. This analysis was moved from the main text to the Supplement at peer review and no conclusion in the paper rests on it.

Chart	Centre line /1,000	Year	Observed /1,000	Limit crossed /1,000	Signal
Neonatal mortality	3.83	2016	6.18	5.87 (upper)	Above upper limit
Infant mortality	4.82	2016	7.66	7.10 (upper)	Above upper limit
Infant mortality	4.82	2024	2.57	2.69 (lower)	Below lower limit
Stillbirth	9.14	2025	5.42	6.14 (lower)	Below lower limit
Antenatal stillbirth	7.58	2025	4.38	4.84 (lower)	Below lower limit
Perinatal mortality	11.41	2025	6.99	8.06 (lower)	Below lower limit

Intrapartum stillbirth (centre line 1.56 per 1,000) produced no signal in any of the eleven years. Given the width of the limits at these counts, that absence indicates limited power to detect a moderate change and is not evidence that the rate was constant.
